# Predicting the final metabolic profile based on the succession-related microbiota during spontaneous fermentation of the starter for Chinese liquor making

**DOI:** 10.1128/msystems.00586-23

**Published:** 2024-01-11

**Authors:** Shibo Ban, Wei Cheng, Xi Wang, Jiao Niu, Qun Wu, Yan Xu

**Affiliations:** 1 Lab of Brewing Microbiology and Applied Enzymology, Key Laboratory of Industrial Biotechnology of Ministry of Education, State Key Laboratory of Food Science and Technology, School of Biotechnology, Jiangnan University, Wuxi, China; 2 Sichuan Langjiu Group Co., Ltd, Luzhou, China; University of California San Diego, La Jolla, California, USA

**Keywords:** liquor fermentation, metabolites, microbial succession, microbiota, modeling

## Abstract

Microbial inoculation is an effective way to improve the quality of fermented foods via affecting the microbiota structure. However, it is unclear how the inoculation regulates the microbiota structure, and it is still difficult to directionally control the microbiota function via the inoculation. In this work, using the spontaneous fermentation of the starter (*Daqu*) for Chinese liquor fermentation as a case, we inoculated different microbiota groups at different time points in *Daqu* fermentation, and analyzed the effect of the inoculation on the final metabolic profile of *Daqu*. The inoculated microbiota and inoculated time points both significantly affected the final metabolites via regulating the microbial succession (*P* < 0.001), and multiple inoculations can promote deterministic assembly. Twenty-seven genera were identified to be related to microbial succession, and drove the variation of 121 metabolites. We then constructed an elastic network model to predict the profile of these 121 metabolites based on the abundances of 27 succession-related genera in *Daqu* fermentation. Procrustes analysis showed that the model could accurately predict the metabolic abundances (average Spearman correlation coefficients >0.3). This work revealed the effect of inoculation on the microbiota succession and the metabolic profile. The established predicted model of metabolic profile would be beneficial for directionally improving the food quality.

**IMPORTANCE:**

This work revealed the importance of microbial succession to microbiota structure and metabolites. Multi-inoculations would promote deterministic assembly. It would facilitate the regulation of microbiota structure and metabolic profile. In addition, we established a model to predict final metabolites based on microbial genera related to microbial succession. This model was beneficial for optimizing the inoculation of the microbiota. This work would be helpful for controlling the spontaneous food fermentation and directionally improving the food quality.

## INTRODUCTION

Most traditional fermented foods are produced by spontaneous fermentation, which plays an important role in the sensory properties of fermented foods (1). The final metabolic profile is important for the quality of fermented foods (2–4). However, the metabolites are determined by the microbial taxa and metabolic traits (5), and many uncharacterized microbial interactions (6, 7). Connecting microbiota structure to the final metabolites is important for controlling the function of microbiota.

The final metabolites of food fermentation are dependent on microbiota in the whole process of fermentation (8). For example, the increase of initial fungal diversity increases the final metabolic diversity of fermentation (9); and the change of dominant microorganisms during fermentation also influences the final metabolites (10, 11). These studies suggest that microbiota at the beginning and during the fermentation would both influence the final metabolites. Therefore, it is important to analyze the effect of microbiota in the whole fermentation on the final metabolic profile of fermentation to control the quality of fermented food.

The microbiota structure is influenced by the succession in the whole fermentation. The succession of the microbiota is determined by the community assembly which includes stochastic and deterministic processes (12). For stochastic process, drift or stochastic dispersal is the main driving force, and the microbiota presents an unorderly succession (13–15). For deterministic process, environmental filtering is the main driving force (16), and the microbiota presents an orderly succession, and is more predictable (17, 18). Deterministic assembly is beneficial for the microbial adaption to the variation of fermentation environment (19), and can accelerate functional microorganisms to become dominant (20). As a result, it is important to reveal the relationship of the structure and succession of the microbiota, and control the microbiota structure via the succession of the microbiota, that would be beneficial for controlling the final metabolites in food fermentation.

*Daqu*, as the starter of Chinese liquor fermentation, is produced by spontaneous fermentation (21, 22). It is suitable as a model system for the study of microbial succession and regulation. The metabolites produced by the microbiota in *Daqu* play important roles in the quality of Chinese liquor (22). As a result, controlling the final metabolites of *Daqu* would be beneficial for controlling the liquor quality. In this work, we separately inoculated different microbiota at different times of *Daqu* fermentation, and studied the effect of inoculation on microbial succession and final metabolites in *Daqu* fermentation. We then established a model to predict the final metabolites based on the succession-related microbiota at different times in *Daqu* fermentation. This work revealed the effect of microbial inoculation on the final metabolites of spontaneous food fermentation, and provided a novel strategy to control the final metabolites in food fermentation. It would be beneficial for improving the quality of fermented foods.

## RESULTS

### Effect of microbial inoculation on microbiota in *Daqu* fermentation

**Fig 1:**
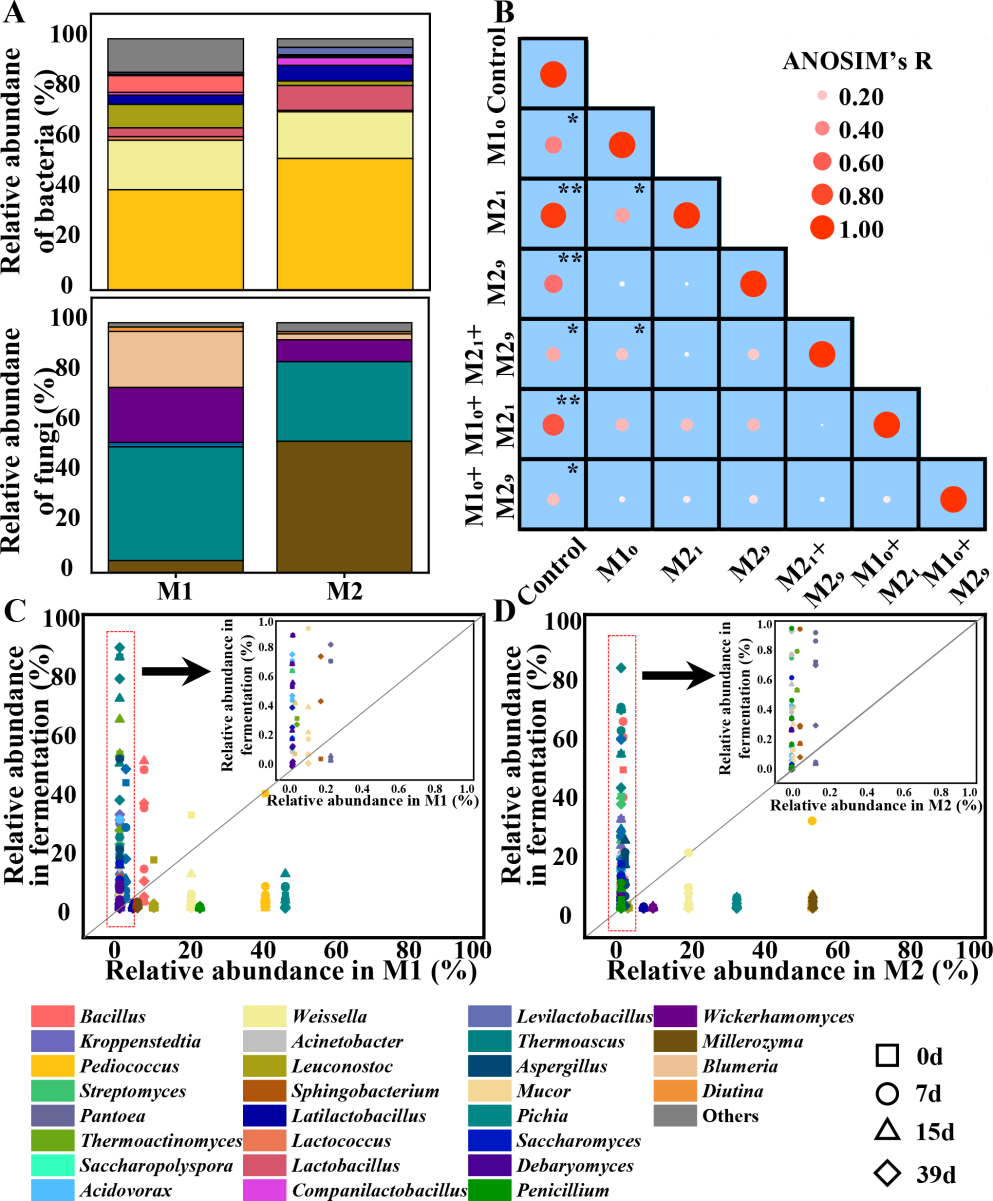
The microbiota characteristics in *Daqu* fermentation after microbial inoculation. (**A**) Relative abundances of bacteria and fungi in microbiota groups M1 and M2. Genera with average abundances greater than 1% are indicated. (**B**) Differences analysis for microbiota in different inoculated groups. The *R*- and *P*-values are calculated by using ANOSIM, ***P < 0.001; **P < 0.01; *P < 0.05. Control, without inoculation. M1_0_, inoculation of M1 on day 0; M2_1_, inoculation of M2 on day 1; M2_9_, inoculation of M2 on day 9; M2_1_ + M2_9_, inoculations of M2 on days 1 and 9; M1_0_ + M2_1_, inoculations of M1 on day 0 and M2 on day 1; M1_0_ + M2_9_, inoculations of M1 on day 0 and M2 on day 9. The colonization of microbial genera from M1 (**C**) and from M2 (**D**) in *Daqu* fermentation. The abundances of genera in microbiota group (M1 or M2) and *Daqu* fermentation are showed on the abscissa and ordinate, respectively. The genera above the diagonal line represent their relative abundances are higher than that in *Daqu* fermentation. The genera with relative abundances lower than or equal to 1% in M1 or M2, are highlighted in the red dotted box.

We cultured the fermented *Daqu* in wheat extract medium to enrich the microbiota during *Daqu* fermentation, and obtained two different microbiota groups (M1 and M2). *Bacillus* (6.70%), *Pediococcus* (40.1%), *Weissella* (19.66%), *Leuconostoc* (9.35%), and *Latiactobacillus* (3.66%) were dominant bacterial genera (relative abundance >1%) in M1. *Pediococcus* (52.48%), *Weissella* (18.51%), *Leuconostoc* (1.83%), and *Latiactobacillus* (6.19%) were dominant bacterial genera in M2. *Thermomyces* (1.70%), *Pichia* (45.51%), *Wickerhamomyces* (22.03%), and *Millerozyma* (4.89%) were dominant fungal genera in M1. *Aspergillus* (1.09%), *Pichia* (31.77%), *Wickerhamomyces* (8.75%), and *Millerozyma* (52.64%) were dominant fungal genera in M2 (Fig. 1A).

We then inoculated these two different microbiota groups M1 and M2 at different time points during *Daqu* fermentation (Fig. S1), including inoculation of M1 on day 0 (M1_0_), M2 on day 1 (M2_1_), M2 on day 9 (M2_9_), M2 on days 1 and 9 (M2_1_ + M2_9_), M1 on day 0 and M2 on day 1 (M1_0_ + M2_1_), M1 on day 0 and M2 on day 9 (M1_0_ + M2_9_). Compared to the fermentation without inoculation, the microbiota in fermentations with the inoculation were all significantly different (ANOSIM: *R* = 0.219–0.926, *P* = 0.006–0.048). In addition, the microbiota after inoculation were all significantly different from each other (ANOSIM: *R* = 0.044, *P* = 0.043). Moreover, the microbiota in fermentation showed significant differences among the inoculation of different microbiota (M1, M2, and M1 + M2, ANOSIM: *R* = 0.372, *P* = 0.004), and among the inoculation at different time points in the fermentation (days 0, 1, and 9, ANOSIM: *R* = 0.967, *P* = 0.007) (Fig. 1B).

We further analyzed the variation of microbial genera from M1 in *Daqu* fermentation (Fig. S2). For bacteria, the predominant bacteria (*Pediococcus* and *Weissella*, relative abundance >10%), and the dominant bacteria (*Bacillus* and *Leuconostoc*, 10% ≥relative abundance >1%) in M1, became dominant in *Daqu* fermentation (*Pediococcus*, 3.23–83.46%; *Weissella*, 1.49–31.75%; *Bacillus*, 1.46–50.15%; and *Leuconostoc*, 1.00–16.50%). In addition, the nondominant bacteria (*Pantoea*, *Thermoactinomyces*, and *Saccharopolyspora*, relative abundance ≤1%) in M1 also became dominant in *Daqu* fermentation (*Pantoea*, 1.59–14.95%; *Thermoactinomyces*, 1.09–64.27%; and *Saccharopolyspora*, 1.17–15.92%). For fungi, the predominant fungi (*Thermomyces* and *Pichia*), and the dominant fungus (*Millerozyma*), became dominant in *Daqu* fermentation (*Thermomyces*, 2.89–49.39%; *Pichia*, 1.31–11.68%; and *Millerozyma*, 1.03–2.14%). Meanwhile, the nondominant fungi in M1 (*Thermoasucs*, *Aspergillus*, and *Mucor*) also became dominant in *Daqu* fermentation (*Thermoasucs*, 2.86–88.78%; *Aspergillus*, 1.35–50.84%; and *Mucor*, 1.15–44.70%) (Fig. 1C).

For the variation of microbial genera from M2 in *Daqu* fermentation (Fig. S2), the predominant genera (*Pediococcus*, *Weissella*, *Pichia,* and *Millerozyma*) in M2 became dominant in *Daqu* fermentation (*Pediococcus*, 1.26–29.75%; *Weissella*, 1.49–5.04%; *Pichia*, 1.18–11.86%; and *Millerozyma*, 1.03–4.44%), and the dominant genera (*Leuconostoc* and *Aspergillus*) became dominant in *Daqu* fermentation (*Leuconostoc*, 1.00–17.66% and *Aspergillus*, 1.00–50.84%). The nondominant genera (*Kroppenstedtia*, *Pantoea*, *Thermoactinomyces*, *Saccharopolyspora*, *Thermomyces*, *Saccharomyces*, and *Debaryomyces*) became dominant in *Daqu* fermentation (*Kroppenstedtia*, 4.90–57.84%; *Pantoea*, 1.43–36.44%; *Thermoactinomyces*, 1.00–64.27%; *Saccharopolyspora*, 1.16–24.92% *Thermomyces*, 2.89–57.47%; *Saccharomyces*, 1.18–15.16%; and *Debaryomyces*, 1.08–8.75%) (Fig. 1D). These results indicated that the microorganisms in the inoculated microbiota could enter and colonized in *Daqu* fermentation.

### Effect of microbial inoculation on final metabolites in *Daqu* fermentation

**Fig 2:**
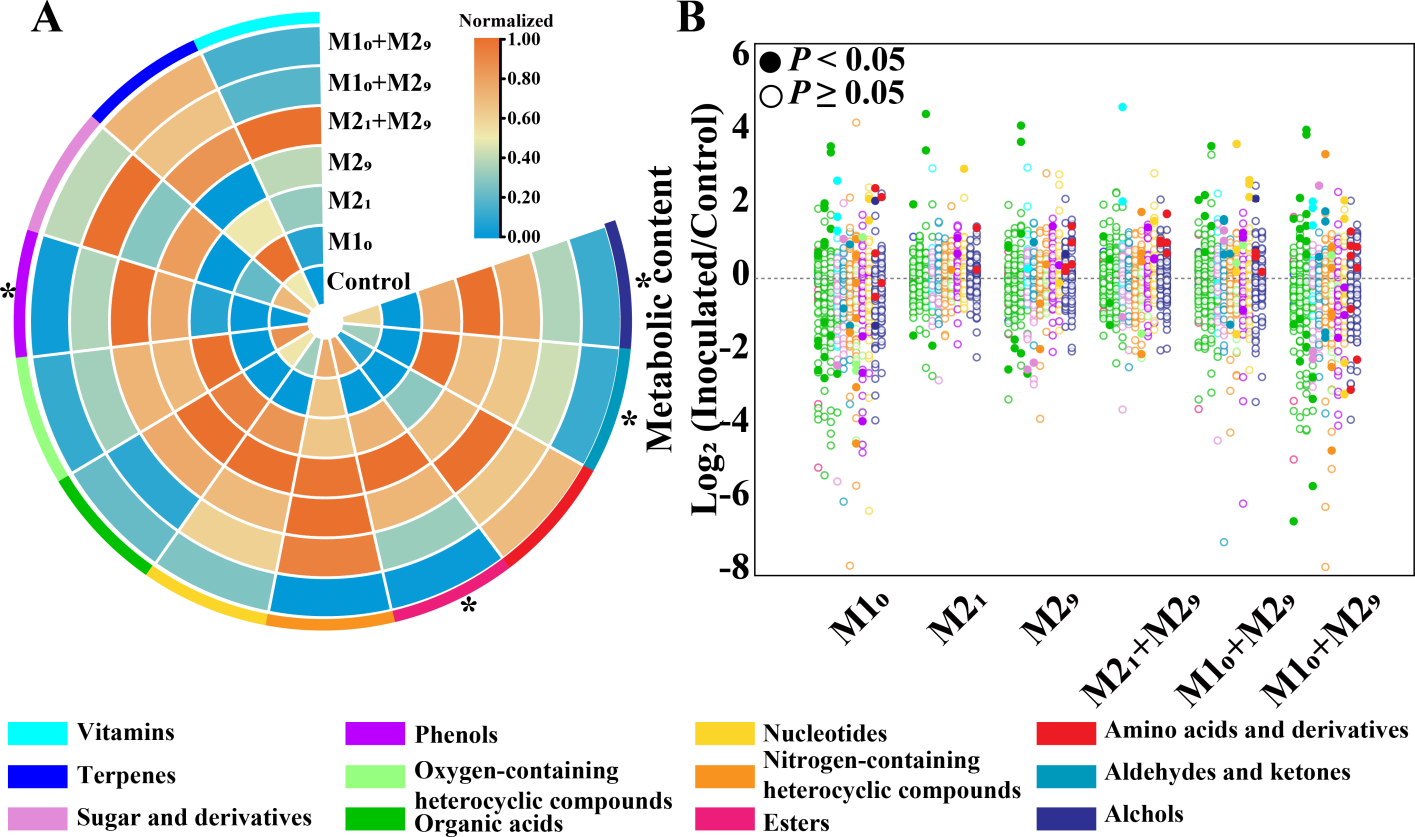
Analysis of final metabolites in *Daqu* fermentation with and without inoculation. (**A**) Heatmap of 12 categories of final metabolites in *Daqu* fermentations. The relative abundances of metabolites are normalized by minimum–maximum normalization. The edge color of each sector indicates the category of metabolites. (**B**) Differential analysis showing varied metabolites across different fermentations. *P* values are calculated with Wilcoxon rank-sum test, solid circles indicate the metabolites with significant differences (*P* < 0.05), and hollow circles indicate the metabolites with insignificant differences (*P* ≥ 0.5). Each circle indicates one metabolite at the end of *Daqu* fermentation. The color of circle indicates the category of metabolites. Solid circles with log_2_ (Inoculated/Control) higher or less than 0 indicate increased and decreased metabolites, respectively. Control, without inoculation; Control, without inoculation; M1_0_, inoculation of M1 on day 0; M2_1_, inoculation of M2 on day 1; M2_9_, inoculation of M2 on day 9; M2_1_ + M2_9_, inoculations of M2 on days 1 and 9; M1_0_ + M2_1_, inoculations of M1 on day 0 and M2 on day 1; M1_0_ + M2_9_, inoculations of M1 on day 0 and M2 on day 9. *P < 0.05.

To analyze the effect of microbial inoculation on final metabolites in *Daqu* fermentation, we determined the metabolites at the end of fermentation. A total of 546 metabolites were obtained (Fig. S3), and these metabolites were classified into 12 categories (Fig. 2A).

Compared to fermentation without inoculation, final metabolic profiles were all significantly different (ANOSIM: *R* = 0.037–0.370, *P* = 0.006–0.015). In addition, the final metabolic profiles after inoculation were all significantly different from each other (ANOSIM: *R* = 0.313, *P* = 0.001). Moreover, the metabolic profiles showed significant differences among the inoculated different microbiota (M1, M2, and M1 + M2, ANOSIM: *R* = 0.188, *P* = 0.048), and among the inoculations at different time points in the fermentation (days 0, 1, and 9, ANOSIM: *R* = 0.337, *P* = 0.005).

Compared to fermentation without inoculation, four categories of metabolites were significantly different (Kruskal-Wallis: phenols, *P* = 0.042; alcohols: *P* = 0.041; esters: *P* = 0.036; aldehydes and ketones: *P* = 0.030) among all the fermentations with the inoculation. The final metabolites varied greatest with the inoculation of M2 on both days 1 and 9, with the number of significantly different metabolites (*P* < 0.05) were highest (88 metabolites), and the abundances of nine categories (alcohols, aldehydes and ketones, amino acids and derivatives, esters, nitrogen-containing and heterocyclic compounds, nucleotides, organic acids, phenols, sugar and sugar alcohols) were significantly different (*P* = 0.027), compared with that without inoculation (Fig. S4).

The variation of final metabolites in fermentations with inoculation was calculated (Fig. 2B; Fig. S4; Table S4). An average of 30 and 16 metabolites were significantly increased (*P* = 0.001–0.047) and decreased (*P* = 0.001–0.049) in all these inoculated fermentations, respectively. The number of metabolites with significantly increased abundances was the highest in fermentation with inoculation of M2 on day 9 (62 metabolites), and it was lowest in fermentation with inoculation of M1 on day 1 (10 metabolites).

### Effect of microbial inoculation on microbial succession in *Daqu* fermentation

**Fig 3:**
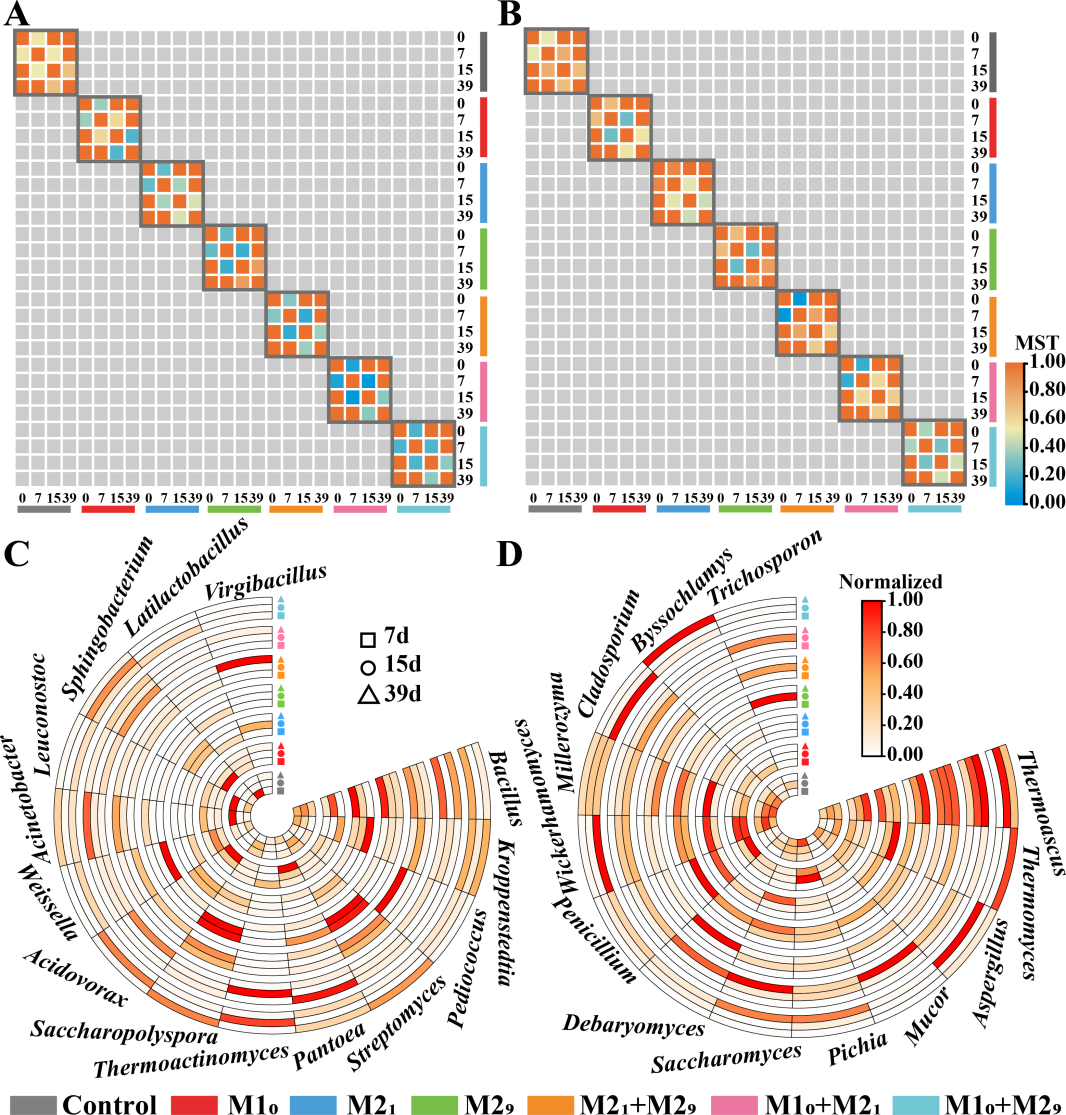
Effect of microbial inoculation on microbial succession in *Daqu* fermentation. The modified stochasticity ratio (MST) value of the bacteria (**A**) and fungi (**B**) in whole fermentation. MST value, with less than 0.5 and greater than 0, indicates deterministic succession of the microbiota; MST value, with less than 1 and greater than or equal to 0.5, indicates stochastic succession of the microbiotas. MST is calculated by comparing a current fermentation time point with that in its previous time point. The abundances of dominant bacterial genera (**C**) and fungal genera (**D**) related to microbial succession. All relative abundances of microbial genera are normalized from 0 to 1 (minimum–maximum normalization), which show the differences in abundances of microbiota on the same day. Control, without inoculation; M1_0_, inoculation of M1 on day 0; M2_1_, inoculation of M2 on day 1; M2_9_, inoculation of M2 on day 9; M2_1_ + M2_9_, inoculations of M2 on days 1 and 9; M1_0_ + M2_1_, inoculations of M1 on day 0 and M2 on day 1; M1_0_ + M2_9_, inoculations of M1 on day 0 and M2 on day 9.

To reveal the effect of microbial inoculation on microbial succession, we calculated modified stochasticity ratio (MST) based on a general framework to assess ecological stochasticity in fermentation. MST is in the threshold between 0 and 1. It is stochastic assembly when MST ≥ 0.5, and is deterministic assembly when MST < 0.5. For bacteria, as shown in Fig. 3A, the bacterial community kept the stochastic assembly (7 days: MST = 0.559, 15 days: MST = 0.540, 39 days: MST = 0.698) in *Daqu* fermentation without inoculation. After the inoculation of M1 on day 0, the succession of bacterial community became deterministic assembly (MST = 0.385) on day 7, but the deterministic assembly cannot be maintained until day 15 (MST = 0.616). When M2 was inoculated on day 1, the bacterial community on days 7 and 15 both became deterministic assembly (7 days: MST = 0.277 and 15 days: MST = 0.409), but it became stochastic assembly (MST = 0.504) at the end of fermentation. When M2 was inoculated on day 9, the bacterial community on day 15 became deterministic succession (MST = 0.200), but it was still stochastic assembly (MST = 0.830) at the end of fermentation. When M2 was inoculated on both days 1 and 9, the bacterial community was deterministic assembly (7 days: MST = 0.343, 15 days: MST = 0.183, 39 days: MST = 0.388) during the whole fermentation. When M1 and M2 were both inoculated, the bacterial community was deterministic assembly (M1_0_ + M2_1_, 7 days: MST = 0.343, 15 days: MST = 0.183, 39 days: MST = 0.388; M1_0_ + M2_9_: 7 days: MST = 0.235, 15 days: MST = 0.240, 39 days: MST = 0.362) during the whole fermentation.

For fungi, as shown in Fig. 3B, the fungal community kept stochastic assembly (7 days: MST = 0.511, 15 days: MST = 0.540, and 39 days: MST = 0.698) in *Daqu* fermentation without inoculation. When the inoculation of M1 on day 0, or the inoculation of M2 on day 1, or the inoculation of M2 on day 9, the succession of fungal community became deterministic assembly (MST = 0.263–0.443) on day 15, but it became stochastic assembly at the end of the fermentation (MST = 0.531–0.827). When the inoculations of M1 on day 0 and M2 on day 1, the succession of fungal community became deterministic assembly on day 7 (MST = 0.057), but it was still stochastic assembly after then in the fermentation (MST = 0.649–0.803). In addition, when the inoculations of M1 on day 0 and M2 on day 9, the succession of fungal community became deterministic during the whole fermentation (7 days: MST = 0.397, 15 days: MST = 0.292, and 39 days: MST = 0.467). These results indicated that the microbial succession shifted to deterministic assembly at the subsequent time when the microbiota group was inoculated at a single time point, but this deterministic assembly process cannot be maintained for a long time. The microbial succession would become deterministic assembly at the subsequent time for a longer time period when the microbiota group was inoculated at two different time points. Therefore, the inoculated microbiota group and inoculated time points both played important roles in microbial succession.

We further analyzed dominant genera related to microbial succession by using Kruskal-Wallis *H* Test (Fig. 3C–D). Among all bacterial genera related to microbial succession, *Kroppenstedtia* (*P* = 0.006), *Acidovorax* (*P* = 0.006), *Weissella* (*P* = 0.011), *Pediococcus* (*P* = 0.013), *Saccharopolyspora* (*P* = 0.032), *Thermoactinomyces* (*P* = 0.035), *Acinetobacter* (*P* = 0.039), and *Sphingobacterium* (*P* = 0.043) were significantly different in bacterial succession in different fermentation groups (Fig. 3C). *Penicillium* (*P* = 0.013), *Thermomyces* (*P* = 0.019), *Mucor* (*P* = 0.023), *Debaryomyces* (*P* = 0.023), *Rhizomucor* (*P* = 0.028), *Trichosporon* (*P* = 0.037), *Aspergillus* (*P* = 0.045), *Cladosporium* (*P* = 0.046), and *Thermoascus* (*P* = 0.049) were significantly different fungal genera related to microbial succession (Fig. 3D). These 27 microbial genera were identified as succession-related microbiota, and could be used to regulate the succession of the microbiota.

### Microbial succession influences final metabolites in *Daqu* fermentation through deterministic assembly

**Fig 4:**
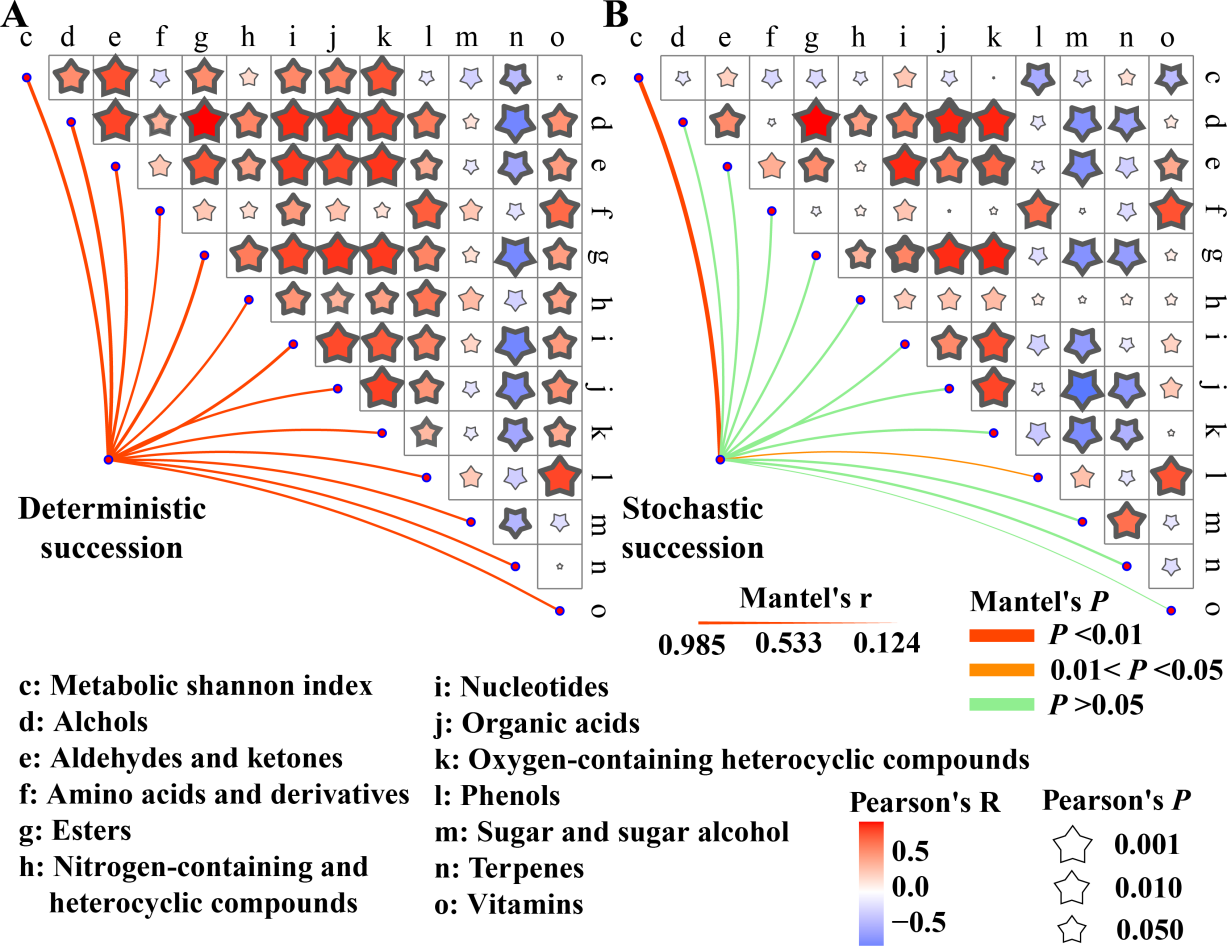
The correlation between microbial succession and final metabolites in *Daqu* fermentation. The effects of deterministic assembly (**A**) and stochastic assembly (**B**) on final metabolic characteristics. The width and color of lines indicate the mantel’s *R*-value and mantel’s *P*-value, respectively. Lines with Mantel’s *P* < 0.05 indicate significant correlations. The size and color of stars indicate the Pearson’s *R*-value and Pearson’s *P*-value, respectively. Bold stars with Pearson’s *P*-value < 0.05 indicate significant correlations.

In order to analyze the effect of microbial succession on final metabolites in *Daqu* fermentation, we constructed the mantel test and correlation matrix. The deterministic assembly was correlated with metabolic Shannon index (*R* = 0.32, *P* < 0.01), abundances of alcohols (*R* = 0.76, *P* < 0.01), aldehydes and ketones (*R* = 0.46, *P* < 0.01), amino acids and derivatives (*R* = 0.26, *P* < 0.01), esters (*R* = 0.84, *P* < 0.01), nitrogen-containing compounds (*R* = 0.79, *P* < 0.01), nucleotide (*R* = 0.57, *P* < 0.01), organic acids (*R* = 0.52, *P* < 0.01), oxygen-containing heterocyclic compounds (*R* = 0.55, *P* < 0.01), phenols (*R* = 0.66, *P* < 0.01), sugar and sugar alcohols (*R* = 0.10, *P* < 0.01), terpenes (*R* = 0.54, *P* < 0.01), and vitamins (*R* = 0.85, *P* < 0.01) (Fig. 4A). However, stochastic assembly was only correlated with the metabolic Shannon index (*R* = 0.98, *P* < 0.01) and abundance of phenols (*R* = 0.33, *P* < 0.05) (Fig. 4B). Driven by deterministic succession, alcohols, aldehydes and ketones, esters, and organic acids were significantly correlated with other metabolites, which were also key flavor compounds of Daqu. However, driven by stochastic succession, the above four categories of metabolites had no high correlation with other metabolites. It suggested that the variation of metabolites was mainly correlated with deterministic assembly, and deterministic assembly presented the main effect on the final metabolites.

### Prediction of final metabolites based on the microbial inoculation in *Daqu* fermentation

**Fig 5:**
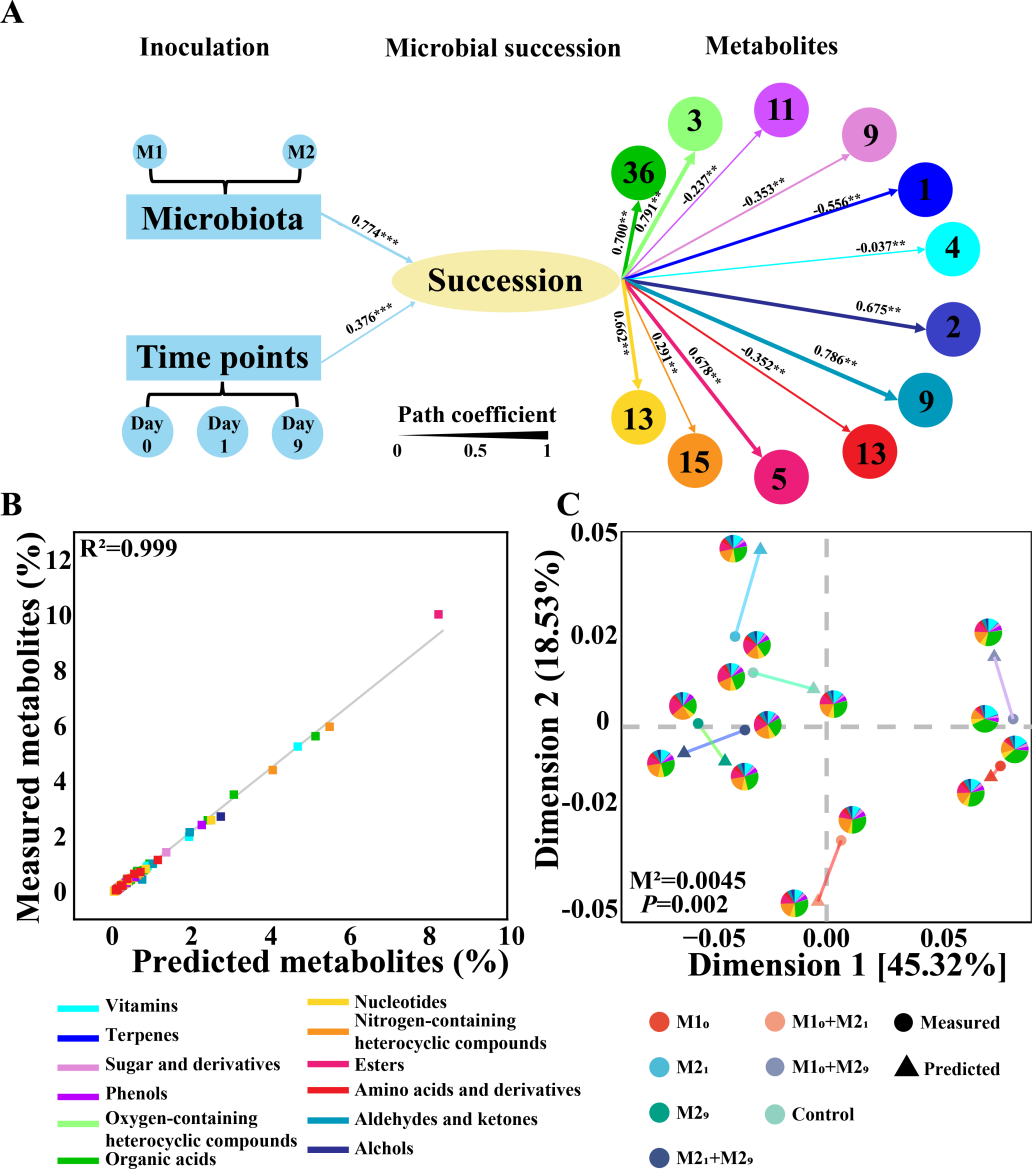
Prediction of final metabolites based on the microbial inoculation in *Daqu* fermentation. (**A**) Structural equation model (SEM) revealing the relationship among microbial inoculation, succession, and metabolites. The width of arrows indicates the strength of path coefficient. The arrows represent path coefficients of standardized regression, and the data along with arrows represent the path coefficient value. *P*-values, determined by Tukey’s test, indicate the significance of the path. ***P < 0.001; **P < 0.01; *P < 0.05. Each metabolite circle indicates a category of metabolites, and the numbers in circle represent the number of metabolites that can be predicted. (**B**) The Spearman correlation coefficient of predicted metabolic abundances and measured metabolic abundances using linear regression analysis. (**C**) The comparison of the metabolic profiles between predicted value and measured value using Procrustes analysis. Each pie represents one metabolic profile classified by metabolic categories. Circles represent measured metabolic values, and triangles represent predicted metabolic values. Lines connecting the circles and triangles indicate the distances between the measured and predicted profiles. Control, without inoculation; M1_0_, inoculation of M1 on day 0; M2_1_, inoculation of M2 on day 1; M2_9_, inoculation of M2 on day 9; M2_1_ + M2_9_, inoculations of M2 on days 1 and 9; M1_0_ + M2_1_, inoculations of M1 on day 0 and M2 on day 1; M1_0_ + M2_9_, inoculations of M1 on day 0 and M2 on day 9.

Structural equation modeling (SEM) was further used to evaluate the relationship among the inoculation, microbial succession, and metabolites (Fig. 5A). The different inoculated microbiota groups and inoculated time points all significantly influenced the microbial succession (microbiota: path coefficient = −0.774, *P* < 0.001; time points: path coefficient = 0.376. *P* < 0.001), and the microbial succession affected all 12 categories of metabolites (path coefficient = 0.237–0.785, *P* < 0.01). A total of 121 compounds could be significantly affected by the microbial succession (*P* < 0.05), and they included 2 alcohols, 9 aldehydes and ketones, 13 amino acids and derivatives, 5 esters, 15 nitrogen-containing and heterocyclic compounds, 13 nucleotides, 36 organic acids, 3 oxygen-containing heterocyclic compounds, 11 phenols, 9 sugar and derivatives, 1 terpene, and 4 vitamins. The results indicated that the inoculation could influence the final metabolites through changing the microbial succession. As a result, we hypothesized that these 121 compounds could be predicted by the inoculation.

We established a model to predict the relative abundances of these 121 metabolites based on the succession-related microbiota (27 succession-related genera, including 14 bacterial genera and 13 fungal genera) at different time points (days 0, 7, and 15). The relative abundances of the metabolites were predicted by MelonnPan through initial filtering, model fitting and internal cross-validation (Tables S1 and S2). The abundances of 121 metabolites can be well predicted (Fig. 5B; Table S1) (Spearman *R* > 0.30, *P* < 0.05). We also compared the predicted relative abundances of the metabolic categories with the Procrustes analysis. It indicated that this model could predict the metabolites accurately (*M*^2^ = 0.0045, *P* = 0.002), and the Spearman correlation of predicted metabolic profile and measured metabolic profile were all higher than 0.6, indicating this model can successfully predict the final metabolites based on the succession-related microbiota at different time points during the fermentation (Fig. 5C). For test the models, we collected three other fermentation samples, predicted microbiota of these fermented samples, and achieved accurate predictions of 93 metabolites, including 1 alcohol, 10 aldehydes and ketones, 15 amino acids and derivatives, 3 esters, 15 nitrogen-containing compounds, 7 nucleotides, 24 organic acids, 8 phenols, 6 sugar and sugar alcohols, 1 terpene, and 3 vitamins (Fig. S5). It indicated that 76.86% of the 121 metabolites were accurately predicted.

## DISCUSSION

In spontaneous fermentation of *Daqu*, the succession of the microbiota is usually stochastic assembly (23). However, the functional genera are expected to be dominant during fermentation, which usually requires a deterministic process (19). Microbial colonization is important for accelerating deterministic assembly (24–26). Therefore, inoculation would be an efficient way to accelerate the deterministic assembly of the microbiota. This study provided a method to accelerate the formation of deterministic assembly by directional microbial inoculation.

The assembly way of the microbiota plays an important role in the microbial metabolism. For the deterministic assembly, the functional genera might become dominant, and would be beneficial for producing more metabolites. We revealed that microbial inoculation influenced the final metabolites through microbial succession. At present, inoculation with a single strain or several strains is usually used to modify the microbiota structure in food fermentations (27–29), but there is usually limited effect on the microbiota structure and the final metabolites, due to the exclusion of indigenous microbiota. Inoculation with the microbiota enriched from indigenous microbiota may lessen this exclusion effect, change the microbial succession, and consequently change the final metabolites. Recently, microbial inoculations were mainly performed at the beginning of food fermentation (27, 30, 31). Although it may affect the microbial structure, we revealed a temporary effect when microbial inoculation was performed at only single time point. Inoculation at more time points would lengthen the effects of the modification of the microbial structure and metabolism, via keeping a longer deterministic process. As a result, inoculation of the microbiota at multiple time points would be an efficient way to improve the microbial succession and metabolism, which can be applied to improve the food quality.

Succession-related microorganisms were always present in the succession and remained dominant. In this study, a total of 27 dominant genera were identified to be succession-related microorganisms. They are not only dominant in one stage, but also transmitted to the next stage in succession. It was reported that *Thermoascus* was a kind of heat-resistant microorganism that played a major role in microbiota succession (32); *Weissella* and *Pediococcus* were also the dominant bacterial genera in *Daqu* fermentation and storage period (33–35). These genera related to microbial succession all contributed to the final metabolic profile, and they should also be considered as core microbiota in food fermentation, rather than only flavor compound producers. In addition, we found that the number of fungi that can significantly change in each group during fermentation was much smaller than that of bacteria, which indicated that the scope of regulation of fungi was less than that of bacteria. We hypothesized that this might be the reason why fungal communities were notably more stochastic than the bacterial communities.

This work established a model to predict the final metabolic compounds based on the independent variables, the microbiota structure at different time points. This is the first predictive model to link the final metabolites and the microbiota at the beginning and during the fermentation. In addition, the final metabolic profile (both the types and the abundances of the metabolites) of 121 metabolites can also be predicted. Besides the prediction of the final metabolic profile via succession-related microbiota at different time points, this model can also be used to direct the inoculation experiment. Further, the model would contribute to optimize the inoculated microbiota and inoculated time points, via inputting the desired metabolic profile. In this work, the predictive model was constructed based on relative abundance of the microbiota and metabolites, quantitative results should be used to train and improve the model in the future (36, 37). In addition, one of the independent variables, the members of the succession-related microbiota, can be reduced to simplify the model, which would make the model more applicable.

These results indicated that the microbiota structure in fermentation determined the differences of microbial succession and final metabolic level, and the microbiota structure in different stages has direct or indirect effects on the microbial abundance in subsequent stages. Therefore, this work provided a foundation for the study of the structure and function of microbiota. The model in this work is suitable for the prediction of metabolites in *Daqu* fermentation, and the prediction of metabolites can be realized based on the succession-related microbiota in more fermentation systems. Furthermore, the fermentation quality corresponding to different succession types should be refined in the future to achieve better prediction of fermentation.

### Conclusion

In this work, we revealed that the microbial inoculation would accelerate the deterministic assembly of the microbiota, which significantly affected the abundance of final metabolites. We built a model to predict the final metabolites based on the succession-related microbiota during *Daqu* fermentation. The model can be used to guide the inoculation of microbiota to improve *Daqu* fermentation. This work would promote the regulation of microbiota in food fermentation, and provide a direction to better control food fermentation.

## MATERIALS AND METHODS

### Experiment description

The experiment was conducted in a liquor distillery in Sichuan province, China (106°10′N, 28°9′E) from May to June in 2020. Production steps of high-temperature *Daqu*: Raw material (wheat) was smashed, mixed with the last batch of *Daqu* and water at a ratio of 13:1:3 (wt/wt/vol). The mixture was pressed as bricks (37 cm × 28 cm × 7 cm) and the bricks were piled up into five layers in a fermentation room (6 m × 3.8 m × 4.0 m) for 39 days’ fermentation. Two microbiota groups (M1 and M2) were separately inoculated in *Daqu* fermentation. About 300 g of sample from day 1 of *Daqu* fermentation was inoculated in 5 L wheat extract medium, and microbiota group M1 was obtained after culturing the sample at 30°C for 10 h. Another 300 g of mixed samples from days 1 and 11 in *Daqu* fermentation (1:1, wt/wt) was inoculated in 5 L wheat extract medium, and microbiota group M2 was obtained after culturing the sample at 30°C for 10 h. For preparation of wheat extract medium, wheat and pure water were mixed (1:2, wt/vol), added with α-amylase (30 U/g), and steamed (105°C) for 45 min; then a same volume of pure water containing α−1, 4-glucohydrolase (300 U/g) was added to the mixture, and saccharified at 60°C for 10 h; after centrifuging at 8,000 × *g* for 10 min, the supernatant was obtained as wheat extract medium, in which the sugar content was controlled to 10 °Bx for usage.

We then inoculated these two different microbiota groups M1 and M2 at different time points during *Daqu* fermentation, including inoculation of M1 (2%, vol/wt) on day 0 (M1_0_), M2 (2%, vol/wt) on day 1 (M2_1_), M2 (2%, vol/wt) on day 9 (M2_9_), M2 (2%, vol/wt) on days 1 and 9 (M2_1_ + M2_9_), M1 (2%, vol/wt) on day 0 and M2 (2%, vol/wt) on day 1 (M1_0_ + M2_1_), M1 (2%, vol/wt) on day 0, and M2 (2%, vol/wt) on day 9 (M1_0_ + M2_9_).

### Sample collection

For fermented samples, we collected three parallel samples (200 g) at different fermentation times (days 0, 7, 15, and 39) of seven different fermentation groups, including fermentation group without inoculation and six inoculated fermentation groups. A total of 84 samples were collected from 21 fermentation rooms. For each sample, 100 g of samples was stored at −80°C for DNA extraction and determination of metabolites, and another 100 g of samples was stored at −20°C for physicochemical analysis.

### DNA extraction, qualification, and sequencing analysis

About 7 g of samples was used to extract total DNA using E.Z.N.A (easy nucleic acid isolation) soil DNA kit (Omega Bio-Tek, Nor-cross, GA). For bacteria, the V3-V4 region of 16S rRNA gene was amplified by using the universal primers 338F and 806R (38). For fungi, the ITS2 region was amplified by using the primers ITS2 and ITS3 (39). PCR program was proceeded, as previously described (40). All barcoded PCR products were subjected to high-throughput sequencing using MiSeq sequencing for 2 × 300 bp paired-end sequencing (Illumina, San Diego, CA). All raw data of MiSeq-generated sequence were processed via QIIME (V.1.8) (41). High-quality sequences were obtained by removing sequences that ambiguous homopolymers < 10, bases < 2, primer mismatches, average quality scores < 20, and lengths (excluding the primer or barcode region) <50 bp. Chimeras were removed by UCHIME software (42). And then trimmed sequences were clustered into operational taxonomic units (OTUs) with 97% sequence similarity by using UPARSE (43). All sequence alignment of bacterial 16S rRNA genes and fungal ITS2 regions were using EzBioCloud (www.ezbiocloud.net) and Central Bureau of Fungal Cultures (www.wi.knaw.nl).

### Extraction of metabolites from samples, liquid chromatography-mass spectrum analysis

For extraction of metabolites, 600 µL methanol (containing 2-amino-3-(2-chloro-phenyl)-propionic acid, 4 mg/L) was added to a 2-mL centrifuge tube containing 0.2 g fermented sample. After vortexing for 30 s, the mixture was added with 100 mg glass beads and then ultrasonically treated for 15 min. After that, the mixture was centrifuged at 4°C (13,400 × *g*) for 10 min, and the supernatant was filtrated by a 0.22-μm-pore-size filter. Eight milliliters of filtrate were used for liquid chromatography-mass spectrum (LC-MS) detection.

The LC analysis was performed on a Vanquish UHPLC System (Thermo Fisher Scientific, USA). Chromatography separation was performed on an ACQUITY UPLC HSS T3 150 × 2.1 mm^2^, 1.8 µm, Waters, Milford, MA) with a solvent flow rate of 0.25 mL/min at a column temperature of 40°C. For LC-ESI (+)-MS analysis, the mobile phases consisted of solvent A (0.1% formic acid in acetonitrile, vol/vol) and solvent B (0.1% formic acid in water, vol/vol). The solvent gradient was as follows: 0–1 min 2% A, 1–9 min linear ramp to 2–50% A, 9–12 min linear ramp to 50–98% A, 12–13.5 min hold at 98% A, 13.5–14 min linear ramp to 98–2% A, and 14–20 min hold at 2% A (44). Mass spectrometric detection of metabolites was performed on Q Exactive (Thermo Fisher Scientific, Waltham, MA) with ESI ion source (45).

The raw data were firstly converted to mzXML format by MSConvert in ProteoWizard software package (v3.0.8789) (46), and processed using XCMS (47) for feature detection, retention time correction, and alignment. In this way, the metabolites were identified by accuracy mass (<30 mg/L) and MS/MS data which were used to carry out the molecular feature alignment (48–50). The robust LOESS signal correction (QC-RLSC) (51) was applied for data normalization, and molecular features present in 100% of the QC samples with relative standard deviations below 30% were selected for further metabolite identification.

### Model construction

We used the sequencing data and experimentally measured metabolic abundance of samples to fit the model. An elastic net regularized regression method was used to identify a minimal set of microbiotas that predict metabolites. The final model for predicting is selected based on cross-validation, and the inappropriate metabolites (Spearman correlation coefficient between measured and predicted metabolic abundances across samples, Spearman *R* < 0.3) were abandoned. Furthermore, the sequence features’ coefficients for well-predicted metabolites were obtained and applied to new microbial data to predict the metabolic profiles. The MelonnPan model was developed as a computational method to predict metabolic profiles from microbiota matrix (36). Finally, the table of predicted metabolic abundances was exported. Melonnpan model was constructed by devtools (version 2.4.5), melonnpan (version 4.7-0), optparse (version 4.7-0), and GenABEL (version 4.7-0) package in R (version 3.6.1) (http://huttenhower.sph.harvard.edu/melonnpan).

### Statistical analysis

Amplicon sequencing data were analyzed using QIIME (V.1.8) (52). Analysis of variance (ANOVA) was calculated by using SPSS Statistics 26 (IBM SPSS Statistics, Chicago, IL) to determine differences between different fermentation groups. Sankey diagram was identified by Hmisc (version 4.7-0), networkD3 (version 0.4), and plotly (version 4.10.2) packages in R studio (version 3.6.1). Kruskal-Wallis *H* test was calculated in SPSS Statistics 26. The mantel test correlation was analyzed using the ggcor packages (version 0.9.8.1), and the statistical analyses (significant differences and Procrustes analysis) were calculated by ANOSIM using vegan package (version 2.6-4) in R studio (version 3.6.1) (53). The mantel test (mantel’ *R*- and *P*-value) was calculated using the vegan package (version 2.6-4) to assess Pearson’s correlation between abundances of metabolites and microbial succession (deterministic assembly and stochastic assembly) in R studio (version 3.6.1). The heatmap of MST and succession-related microbiota was figured using TBtools (version 1.108). The relationships between inoculation conditions, microbial succession and metabolites were calculated by establishing SEM using AMOS 21 in SPSS Statistics 26.

## Data Availability

Bacterial and fungal raw sequence data were deposited in the National Microbiology Data Center (NMDC) and NCBI Sequence Read Archive (SRA) databases under the accession numbers NMDC10018437/NMDC10018438 and PRJNA1053892/PRJNA1053895, respectively.

## SUPPLEMENTAL MATERIAL

Fig. S1 to S5 msystems.00586-23-s0001.docx Microbial and metabolic data.

Tables S1 to S4 msystems.00586-23-s0002.xlsx Microbial and metabolic matrices for training models.

ASM does not own the copyrights to Supplemental Material that may be linked to, or accessed through, an article. The authors have granted ASM a non-exclusive, world-wide license to publish the Supplemental Material files. Please contact the corresponding author directly for reuse.
