# Supplementary material for: Predicting the final metabolic profile based on the succession-related microbiota during spontaneous fermentation of the starter for Chinese liquor making
Source: mSystems. 2024 Jan 11;9(2):e00586-23. doi: 10.1128/msystems.00586-23 (PMC10878095; doi:10.1128/msystems.00586-23)
Supplement: Fig. S1 to S5 — Microbial and metabolic data. [file msystems.00586-23-s0001.docx]

**Predicting the final metabolic profile based on the succession-related microbiota during spontaneous fermentation of the starter for Chinese liquor making**

Shibo Ban^1^, Wei Cheng^2^, Xi Wang^2^, Jiao Niu^2^, Qun Wu^1, *^, Yan Xu^1^

^1^ Lab of Brewing Microbiology and Applied Enzymology, Key Laboratory of Industrial Biotechnology of Ministry of Education, State Key Laboratory of Food Science and Technology, School of Biotechnology, Jiangnan University, Wuxi 214122, China.

^2^ Sichuan Langjiu Group Co., Ltd. Luzhou 610213, China.

***Corresponding author:**

Lab of Brewing Microbiology and Applied Enzymology, Key Laboratory of Industrial Biotechnology of Ministry of Education, State Key Laboratory of Food Science and Technology, School of Biotechnology, Jiangnan University, Wuxi 214122, China.

Phone: +86-510-85864112

Fax: +86-510-85864112

E-mail: wuq@jiangnan.edu.cn (Qun Wu)

**Running title:** Microbial succession affects final metabolites


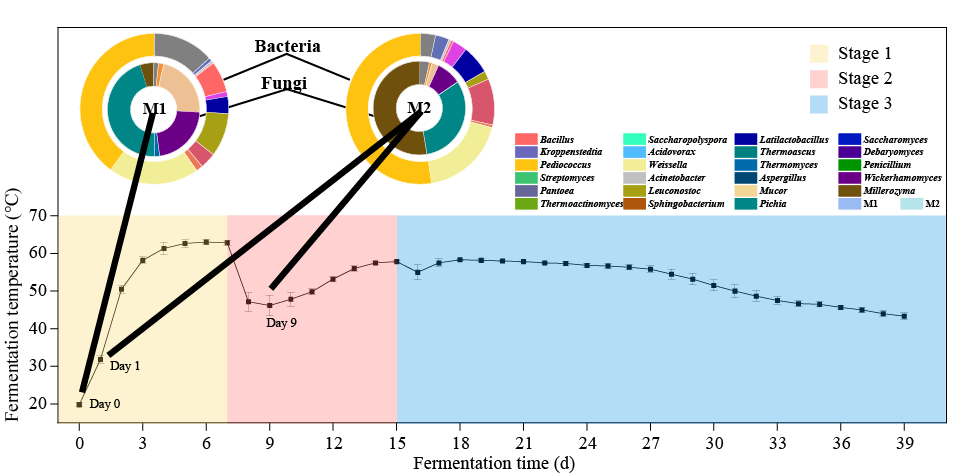


Fig. S1 Inoculation of the microbiota at different time points in Daqu fermentation. Daqu fermentation was classified to three stages based on fermentation temperature, and the microbiota M1 was inoculated on day 0, and the microbiota M2 was inoculated on day 1 and day 9. The circles represent the microbial structure of microbiota M1 and M2, and the outer and inner rings represent the bacterial and fungal community, respectively. *Daqu* was divided into 10 regions (each region about 96 *Daqu*), using pipelines to transport microbiota groups by regions. In the fermentation of 1d and 9d, microbiota groups were directly sprayed in the upper layer, the lower *Daqu* was used pipe transportation.


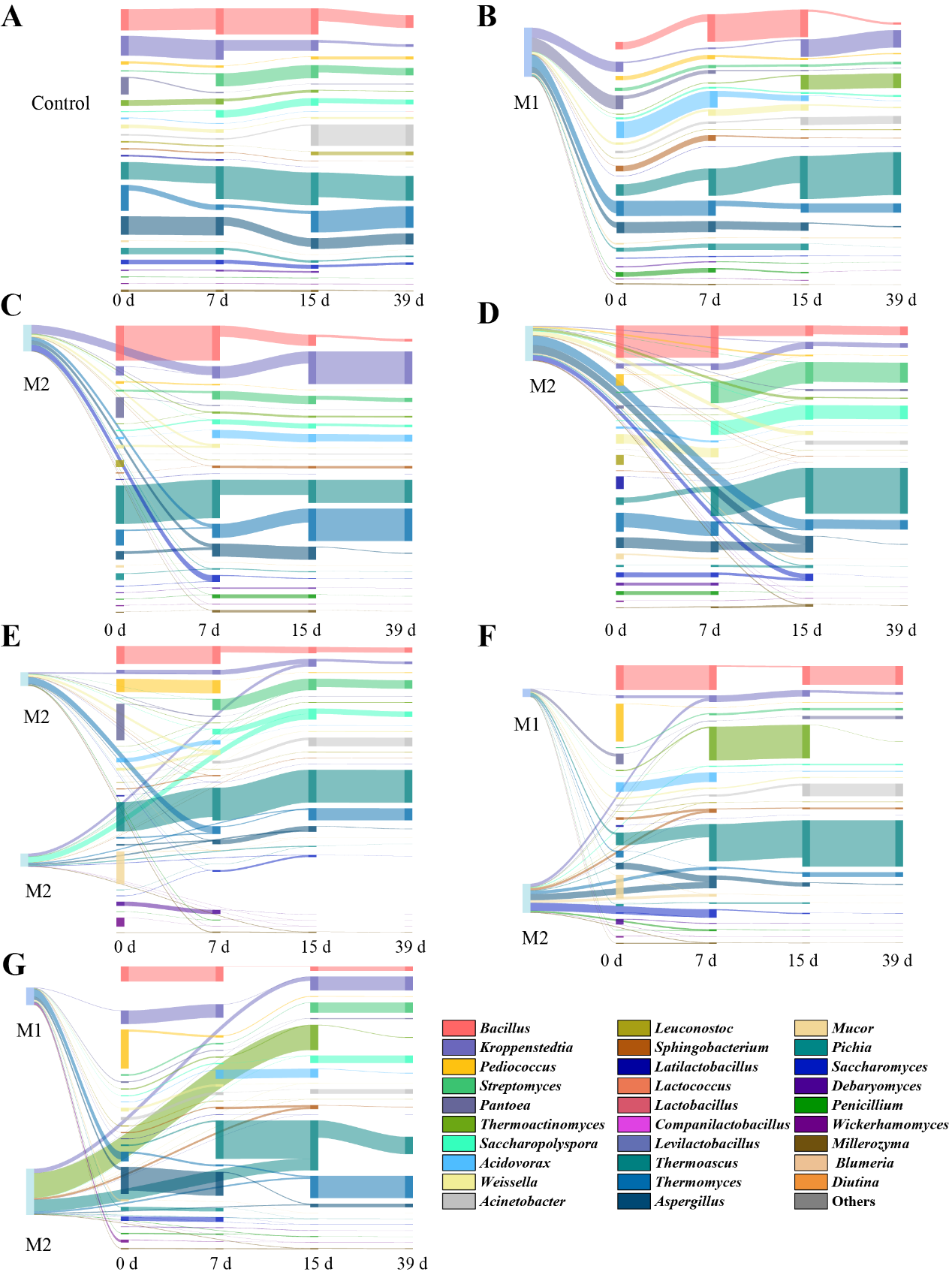


Fig. S2 The abundance of microbiota during fermentation. M1 and M2 represent the microbiota groups. (A) Control, without inoculation; (B) M1_0_, inoculation of M1 on day 0; (C) M2_1_, inoculation of M2 on day 1; (D) M2_9_, inoculation of M2 on day 9; (E) M2_1_+M2_9_, inoculations of M2 on day 1 and day 9; (F) M1_0_+M2_1_, inoculations of M1 on day 0 and M2 on day 1; (G) M1_0_+M2_9_, inoculations of M1 on day 0 and M2 on day 9. The time below each graph indicates fermentation time.


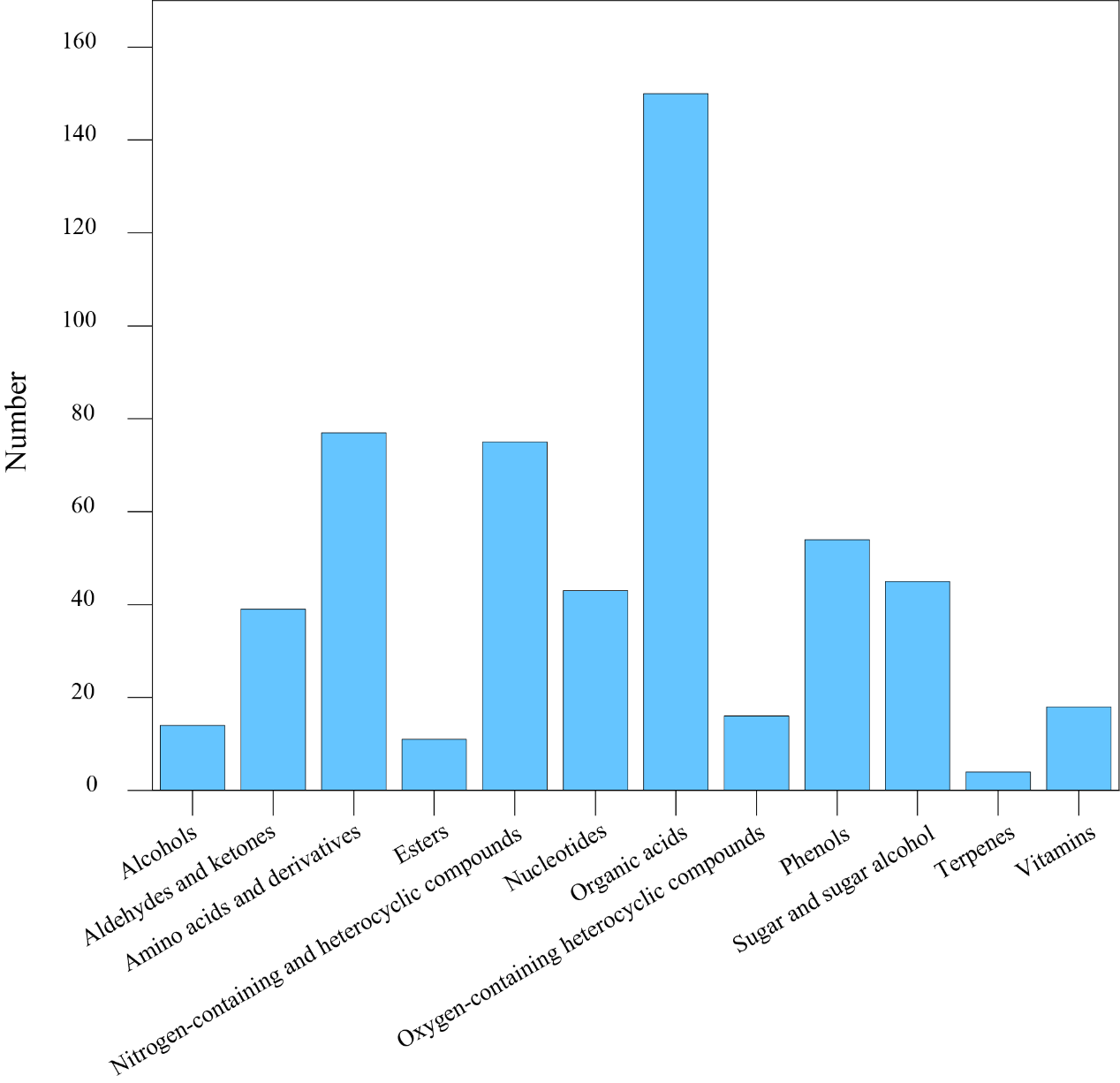


Fig. S3 The number of metabolites in each metabolic category.


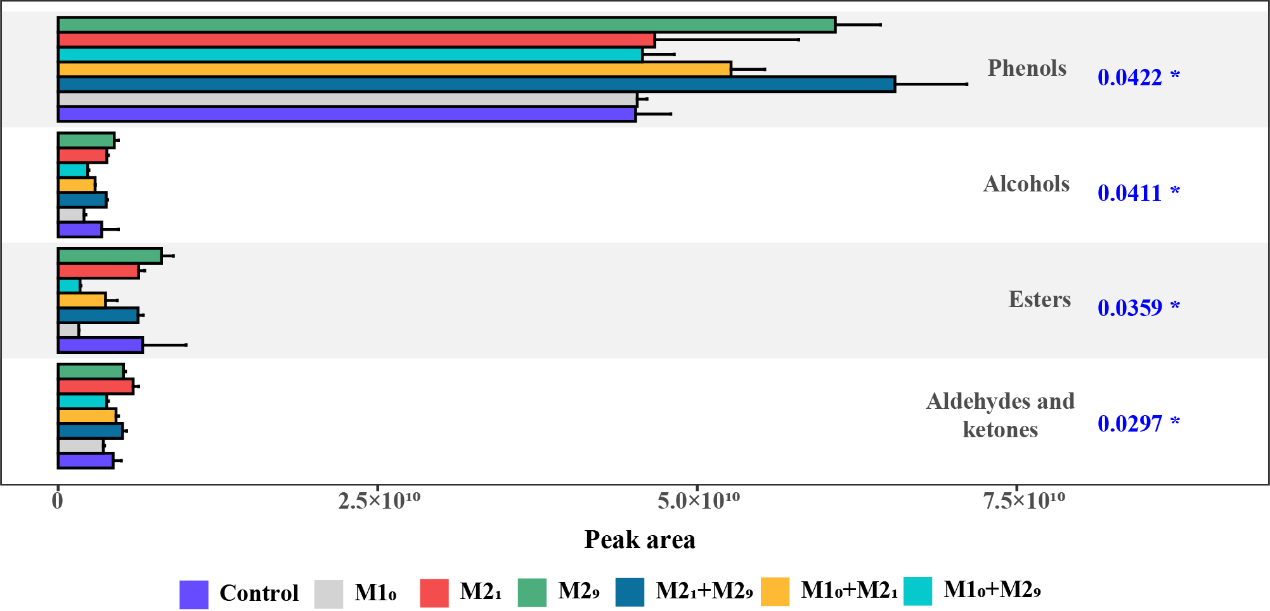


Fig. S4 Analysis of metabolic difference between different groups based on Kruskal-Wallis test. The peak is the raw data for each metabolite in the metabolome data. ****P*<0.001; ***P*<0.01; **P*<0.05; the *P*–values, determined by Tukey’s test. Control, without inoculation; M1_0_, inoculation of M1 on day 0; M2_1_, inoculation of M2 on day 1; M2_9_, inoculation of M2 on day 9; M2_1_+M2_9_, inoculations of M2 on day 1 and day 9; M1_0_+M2_1_, inoculations of M1 on day 0 and M2 on day 1; M1_0_+M2_9_, inoculations of M1 on day 0 and M2 on day 9.


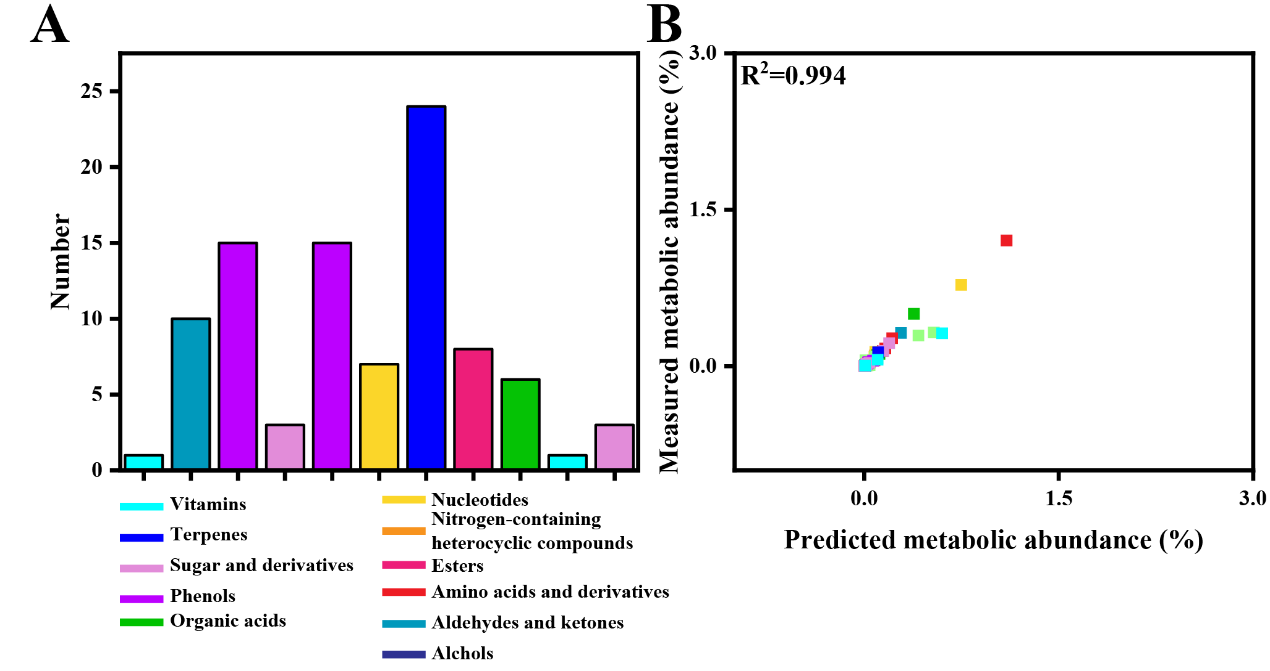


Fig. S5 Prediction of final metabolites based on test data. (A) The number of predicted metabolites and categories. (B) The Spearman correlation coefficient of predicted metabolic abundances and measured metabolic abundances using linear regression analysis.
